# Public Trauma after the *Sewol* Ferry Disaster: The Role of Social Media in Understanding the Public Mood

**DOI:** 10.3390/ijerph120910974

**Published:** 2015-09-03

**Authors:** Hyekyung Woo, Youngtae Cho, Eunyoung Shim, Kihwang Lee, Gilyoung Song

**Affiliations:** 1 School of Public Health, Seoul National University, Seoul 151-742, Korea; E-Mails: hkwoo@snu.ac.kr (H.W.); sey@snu.ac.kr (E.S.); 2 Institute of Health and Environment, Seoul National University, Seoul 151-742, Korea; 3 Mining Laboratory, Daumsoft, Seoul 140-887, Korea; E-Mails: leekh@daumsoft.com (K.L.); kysong@daumsoft.com (G.S.);

**Keywords:** *Sewol* ferry disaster, public mood, public trauma, social media, twitter

## Abstract

The *Sewol* ferry disaster severely shocked Korean society. The objective of this study was to explore how the public mood in Korea changed following the *Sewol* disaster using Twitter data. Data were collected from daily Twitter posts from 1 January 2011 to 31 December 2013 and from 1 March 2014 to 30 June 2014 using natural language-processing and text-mining technologies. We investigated the emotional utterances in reaction to the disaster by analyzing the appearance of keywords, the human-made disaster-related keywords and suicide-related keywords. This disaster elicited immediate emotional reactions from the public, including anger directed at various social and political events occurring in the aftermath of the disaster. We also found that although the frequency of Twitter keywords fluctuated greatly during the month after the *Sewol* disaster, keywords associated with suicide were common in the general population. Policy makers should recognize that both those directly affected and the general public still suffers from the effects of this traumatic event and its aftermath. The mood changes experienced by the general population should be monitored after a disaster, and social media data can be useful for this purpose.

## 1. Introduction

On 16 April 2014, the ferry *Sewol*, which was carrying 476 people including 325 high school students on a school trip, capsized and sank off the southwestern coast of South Korea. This disaster left more than 300 people dead, injured, or missing. The sinking of the *Sewol* severely shocked Korean society. Since the accident, it has been suggested that the public can be traumatized by indirect exposure to certain events through various media [1]. In fact, the scene in which the ferry capsized and sank as crew members were saved, leaving most passengers on board, was broadcast live. The public was repeatedly exposed to this scene for several weeks. 

Early studies have consistently found that a disaster can lead to substantial mental health consequences, including post-traumatic stress disorder (PTSD). However, most of what is known about the mental health consequences of disasters has been derived from studies of focal groups of individuals who were directly exposed to the trauma, such as victims, their families, rescue/recovery workers, volunteers, and the communities in which they live [2]. Relatively few empirical studies have examined the effects of a major disaster on the mental health of the general population. At the same time, interest in public mental health has increased since the 11 September 2001 terrorist attack in New York City. Most data in this domain are derived from studies assessing the reactions of the general public in the US since the September 11 attacks [3,4]. These studies provide evidence of an association between indirect exposure to disaster through media and short-term PTSD-like symptoms [3]. This association was identified by analyzing data from representative samples and retro/prospectively collected social survey data. The data collection and assessments of mental health effects were performed several months to many years after the disaster. This lapse of months or years may cause biases in psychological research because retrospective studies are influenced by recall bias and the emotional state at the time of assessment [2,5]. Therefore, these approaches are not effective ways to monitor public mental health for purposes of real-time surveillance or intervention. 

Accumulating evidence regarding psychological sequelae and the mechanisms associated with the emotional modulation of cognition suggest that vulnerability to disruptions in emotional equilibrium may be a common denominator of mental disorders [6]. It is therefore reasonable to assume that moods, long-term patterns of emotional states, can reflect mental health. In Korea, which is characterized by a consumer economy, the public mood was reflected in the substantial reduction in consumption following the *Sewol* ferry disaster [7]. Although consumer behaviors are among the most meaningful indirect indicators of the public mood [8], ways to directly monitor this mood would be preferable. Recently, several studies have suggested new methods for measuring public mood using social media data. It has been suggested that the analysis of social media data, such as weblog texts or documents, may be a useful way to identify the public mood [8]. 

This study presents a pragmatic simple method for monitoring the public mood using social media data, especially Twitter. This approach may be a better way to identify the post-disaster emotional reactions in the general population in that it permits tracking of public moods through the use of Twitter data. We use Twitter data to explore how Koreans’ public mood changed following the *Sewol* disaster and offer suggestions based on our findings. 

## 2. Methods

### 2.1. Data Sources 

**Figure 1:**
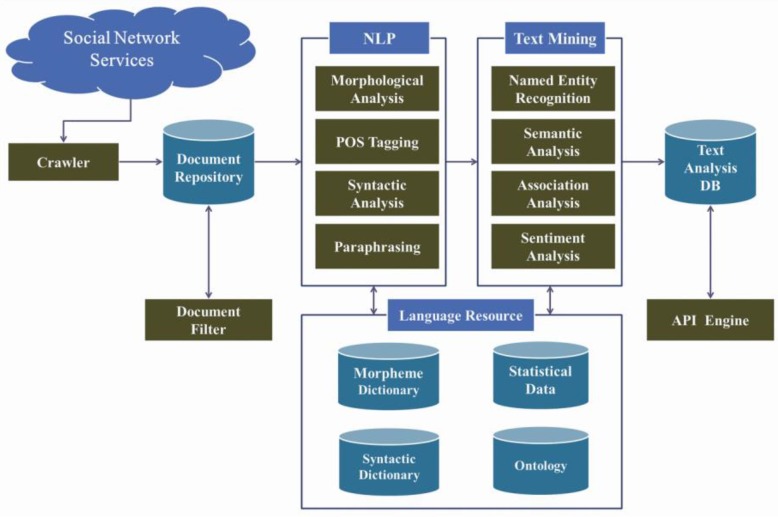
Overall structure of the SOCIALmetrics™ Social Big Data Mining Platform.

Social media data were collected from daily Twitter posts from 1 January 2011 to 31 December 2013 and from 1 March 2014 to 30 June 2014 using the social media analysis tool, SOCIALmetrics™(Daumsoft, Seoul, Korea)./ The SOCIALmetrics^TM^ system contains social media data crawlers that collect posts from Twitter. The system also processes text using state-of-the-art natural language processing (NLP) and text mining technologies (Figure 1).

The NLP module divides input text into sentences and segments the word forms contained in each sentence into a string of morphemes. The segmented morphemes are grouped into syntactic units via syntactic analysis. Once syntactic units are constructed, expressions denoting named entities such as people, locations, and organizations are recognized. Then, association analysis is performed to identify tuples of *<topic keyword, associated keyword>*. Finally, sentiment polarities for topic keywords are determined through sentiment analysis. The results of the whole analysis are delivered in a time-series fashion using an application programmer’s interface (API) engine to accommodate various queries from users. The SOCIALmetrics^TM^ system provides one of the most advanced solutions for the Korean language crawling and mining. Unlike English, Natural Language Processing in Korean is much more complicated. This is due to the fact that the Korean language exhibits characteristics of an agglutinative language and thus there has to be more than one morpheme in order to form a phrase. In the case of the English language, one morpheme is not separated as each word contains a single morpheme; however, the complexity of the Korean language is especially high as morphemes that construct a phrase have to be separated and each morpheme’s part of speech also has to be distinguished. In addition, a Korean word or phrase can carry a very different meaning when used in different linguistic contexts. In order to solve these challenges, SocialMetrics^TM^ utilizes an extensive semantic classification dictionary that contains over 1 million words. The morpheme and phrase analysis used and developed by SOCIALmetrics^TM^ applies a technological method that extracts keywords, going beyond the process of merely selecting simple words. The Twitter crawler utilizes a streaming API [9] for data collection using the so-called “track keywords” function. We tracked several thousand keywords that were empirically selected and tuned to maximize the coverage of the crawler operating in near real-time fashion. We estimated that the daily coverage of the Twitter crawler was over 80%. The collected posts were fed into a spam-filtering module that checks for posts containing spam keywords related to pornography, gambling and other advertising. The lists of spam keywords and spammers were semi-automatically monitored and managed. There is no information that could potentially reveal the identity of social media user, namely user confidentiality is maintained.

### 2.2. Keyword Selection 

#### 2.2.1. Human-Made Disaster-Related Keywords 

A traumatic event elicits a range of negative emotional reactions, including anger, anxiety, and sadness [10,11]. Emotional reactions following human-made disasters tend to be more focused on anger because there are policies and people to blame [11,12]. The *Sewol* disaster was a human-made accident; indeed, the President apologized, the Prime Minister resigned, and all crew members were arrested. Thus, we examined the emotional utterances in reaction to the disaster by analyzing the appearance of three words, “anger,” “anxiety,” and “sadness” on Twitter, focusing especially on anger.

#### 2.2.2. Suicide-Related Keywords

The population-level suicide risk after disasters may be estimated by tracking the specific mood states associated with suicide using social media data. One previous study suggested that specific variables such as suicide-related and dysphoric weblog entries are significantly associated with national suicide rates [8]. The specific mood states associated with suicide can be examined by identifying the emotional words that usually appear with the word “suicide.” We investigated the emotional words most likely to be associated with the Korean word *jasal* (suicide) and *wooul* (depression) using the accumulated tweets submitted to Twitter during the past three years as our database (1 January 2011, to 31 December 2013). Thus, association analysis was performed to identify tuples of topic keyword and associated keyword. Depression-related words were considered along with suicide-related words because depression, similar to PTSD, increases distress and dysfunction over time following traumatic events [13]. Additionally, it is well known that depression is a major risk factor for suicide. 

### 2.3. Generating the Keywords Time Series 

Based on the human-made disasters-related keywords and suicide-related keywords, we generated the keyword time series, defined as the daily volume of tweets mentioning these keywords. First, we processed texts collected from Twitter using state-of-the-art natural language-processing (NLP) and text-mining technologies. The NLP module divides input text into sentences and segments the word forms contained in each sentence into a string of morphemes. Second, the segmented morphemes were grouped into syntactic units via syntactic analysis. Finally, the volumes were analyzed for every 100,000 daily Twitter posts mentioning the Korean words *hwa* (including *bunno*) (anger), *bulan* (anxiety), *seulpeum* (sadness), *chunggyeok* (shock), *seuteureseu* (stress), *gotong* (suffering), *bigeuk* (tragedy), *bulan* (anxiety), *jeolmang* (despair), *bunno* (anger) and *apeum* (pain/hurt) in a time-series fashion. All these volumes were normalized. 

## 3. Results

**Figure 2:**
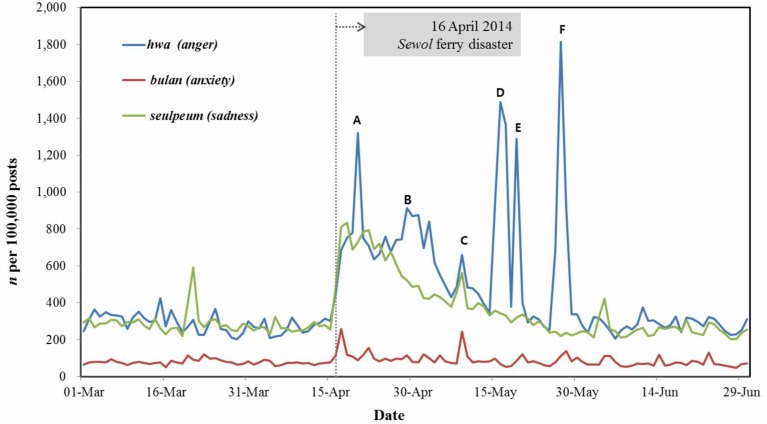
The *Sewol* ferry disaster and negative emotional reactions. (**A**) 20 April, the government declared the affected area a disaster zones. (**B**) 29 April, South Korean president Park Geun-hye visited the memorial alter for victims of the sunken *Sewol* ferry. The death toll surpassed 200 as the *Sewol* search intensified. (**C**) 09 May, There were reports that the sunken *Sewol* ferry was beginning to collapse. (**D**) 17 May, Nationwide rallies in Seoul held to protest government response to ferry sinking. (**E**) 19 May, President Park Geun-hye apologized for the sinking of the *Sewol* during an address to the nation. (**F**) 26, 27 May, Critical rumors about the government response to *Sewol* disaster was rapidly spread abroad on SNS and other media. These were the most widely re-tweeted on Twitter.

Public mood trends were based on daily tweets that reflect public responses to the South Korea ferry disaster. Figure 2 shows the process by which negative emotions unfolded, comparing comments before and after the *Sewol* ferry disaster. The disaster was immediately followed by emotional reactions on the part of the public, with expressions of anger and sadness substantially increasing following the disaster compared with the rates before the disaster. The number of posts mentioning anger and sadness sharply increased during the five days after the disaster. Even though the frequencies of these emotional words gradually decreased after 20 March 2014, their levels during the month following the disaster were notably higher than at baseline. In particular, the number of posts mentioning anger was much higher than were those mentioning anxiety and sadness during the tracking period. Furthermore, expressions of anger rapidly and sharply increased again when specific events related to the disaster occurred. The peak dates (A–F) for anger and brief descriptions of the most important events are included in the figure below (Figure 2).

**Figure 3:**
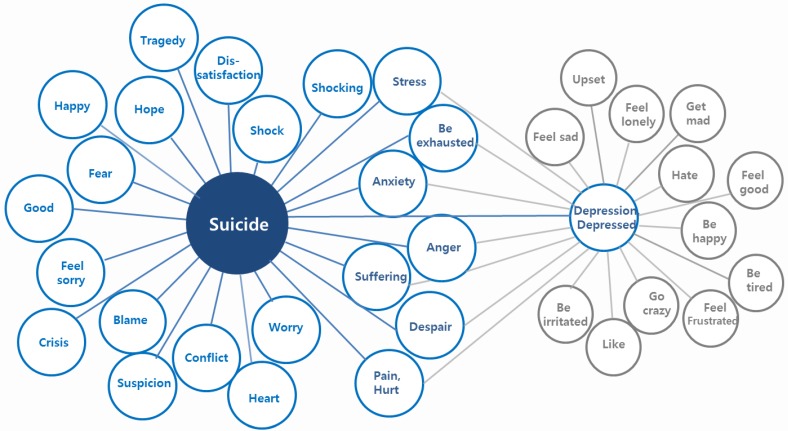
Emotional words most frequently associated with suicide. This diagram shows the words most associated with *jasal* (suicide) and/or *wooul* (depression) during three years. Individual words inside the blue circle are the words associated with suicide, and the word “Depression” is one of these words. The link is defined by association. These data were collected from daily Twitter posts between January 2011 and December 2013.

The suicide-related keywords, we identified by association analysis, are presented in Figure 3. The suicide-related keywords include several emotional words, such as *chunggyeok* (shock), *seuteureseu* (stress), *gotong* (suffering), *bigeuk* (tragedy), *bulan* (anxiety), *jeolmang* (despair), *bunno* (anger), and *apeum* (pain/hurt) (Figure 3). 

**Figure 4:**
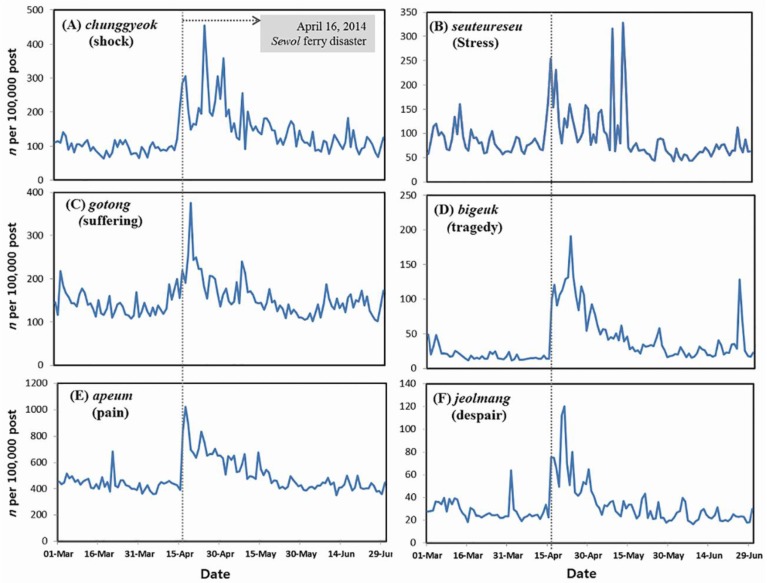
The Sewol ferry disaster and suicide-related public postings.

Figure 4 shows the trends in suicide-related words other than anger and anxiety in the general population before and after the *Sewol* ferry disaster. *Chunggyeok* (shock), *seuteureseu* (stress), *gotong* (suffering), *bigeuk* (tragedy), *bulan* (anxiety), *jeolmang* (despair), *bunno* (anger) and *apeum* (pain/hurt) were the target keywords most frequently associated with *jasal* (suicide) and *wooul* (depression) among the millions of tweets submitted to Twitter during the past three years (1 January 2011 to 31 December 2013). Surprisingly, the disaster led to immediate reactions in terms of suicide-related postings. The frequencies of all suicide-related keywords fluctuated greatly during the month following the disaster. Although we observed distinct differences in the emotional dynamics over time, the levels of all emotions were much higher during the month following the disaster than at baseline (Figure 4).

## 4. Discussion

### 4.1. Human-Made Disasters and Negative Emotional Reactions 

We have found that the *Sewol* ferry disaster caused negative emotional reactions of the public. The pattern of a short-term negative emotional reaction to a human-made disaster followed by its gradual attenuation is generally consistent with previous research findings. Those studies documented a gradual decline over the course of a few months in the PTSD-like symptoms or other stress reactions among members of the general population who experienced the trauma indirectly through media reports [4,5,14]. Additionally, in the early period, the number of posts referring to anger was much higher than those of posts referring to sadness and anxiety, which is also consistent with previous studies of human-made disasters [11,12]. We also found that public anger was easily provoked by various events that occurred in the aftermath of the disaster, such as a report on the exacerbation of the tragedy by the government’s incompetence, which elicited a large-scale reaction. These findings suggest that the dynamics of emotional arousal and coping in a general population after a disaster can be identified through real-time monitoring of specific emotional words appearing on social networking services such as Twitter.

### 4.2. The Sewol Ferry Disaster and Suicide-Related Postings

There is no a general consensus regarding the relationship between disasters and suicide risk. Moreover, most studies of suicide in the aftermath of disasters have focused on natural disasters [15,16], although one study of the aftermath of September 11 found no significant effect of the disaster on the suicide rate of the general population [17]. Links between traumatic events such as human-made disasters and national suicide risk require further research. 

The finding of this study suggests that, at least in Korea, where the suicide rate is generally high, a human-made disaster can lead to an immediate increase in the suicidal preoccupation of the general public. Given that one of the most striking features of contemporary Korean society is its high and increasing suicide rate [18], our findings may have major implications for the national suicide risk in South Korean society after the *Sewol* ferry disaster. It is clear that the use of social media data to identify the moods most likely associated with suicide can be a much faster and easier approach than traditional methods for estimating the suicidality of the general public after disasters or traumatic events.

### 4.3. Lessons, Possibilities, and Further Challenges

Even people who are not directly involved in a disaster may nonetheless be affected by it through various channels, such as repeated news reports on the disaster on television or other media. Policy makers need to remember that the general population does not emerge unscathed from traumatic events, and the aftermath of these events should be the target of monitoring and intervention. Previous studies have noted the challenges to identifying the common characteristics of those affected by a disaster [5]. Historically, people directly associated with disasters were considered vulnerable, but this study suggests that everyone in society may be vulnerable to such events. 

Social media such as Twitter, blogs, and Facebook can be venues for the expression of personal emotions. Once accurate filters and classifiers are developed, these media offer novel opportunities for policy makers to monitor the mental health of the general population by tracking the public mood at any time by analyzing posted texts. Although an initial and well-known example of utilizing social media data for gauging the public mood came from the prediction of box office receipts [19] and stock markets [20], this methodology is being applied in various health-related research fields by tracking the usage of keywords among users of social media services, such as estimating general happiness/subjective well-being [21], influenza outbreaks, [22] and national suicide numbers [8]. Our recent findings have implications for improving research regarding moods and mental health following disasters. Tracking public moods through social media as well as self-assessments of mental states using surveys or physician-reported health records can provide links between traumatic events and mental health responses. Furthermore, it may be more pragmatic to use social media to monitor public mental health for purposes of real-time surveillance or intervention than to use these traditional means. This approach is more immediate and efficient in terms of cost and time than are conventional approaches relying on surveys. Moreover, information from data collected continuously rather than from cross-sectional or sequential designs is more useful for both understanding public mood generation over time and identifying the major determinants of changes in the public mood after traumatic events. Ultimately, these approaches may help policy makers or government agencies find better ways to treat negative public mood or prevent suicide. 

In terms of the future, there is no doubt that social media data can play a foundational role as information sources regarding public health [23,24]. We also suggest that social media data regarding the emotions, thoughts, and desires of individuals offer opportunities to monitor public moods and perspectives through which the mental health and moods of the public can be understood. However, the following issues required additional clarification: *“How close to the truth are the data provided by social media?” and “How do we make social media data more dependable?”* That is, the development of empirical justifications for knowledge derived from social media and the design of sophisticated methodologies for analyzing data derived from social media are challenges for the future.

## 5. Conclusions

Policy makers should recognize that both those directly affected and the general public still suffers from the effects of this traumatic event and its aftermath. The mood changes experienced by the general population should be monitored after a disaster, and social media data can be useful for this purpose. 
